# Emotional Intelligence and Psychological Well-Being of Turkish Physical Education and Sports Athlete–Students: The Mediating Roles of Self-Efficacy and Burnout

**DOI:** 10.3390/bs15030314

**Published:** 2025-03-05

**Authors:** Demet Öztürk Çelik

**Affiliations:** Department of Physical Education and Sports Teaching, Osmaniye Korkut Ata University, 80000 Osmaniye, Türkiye; demetozturk@osmaniye.edu.tr; Tel.: +90-328-827-1000

**Keywords:** emotional intelligence, psychological well-being, self-efficacy, burnout, physical education and sports

## Abstract

The psychological well-being of athlete–students during higher education is important in their healthy growth and adaptation to college and social life, and emotional intelligence is one of the key factors linked with psychological well-being. The present study aimed to investigate the relationship between emotional intelligence and psychological well-being among Turkish athlete–students in higher education studying physical education and sports. A total of 226 athlete–students studying physical education and sports participated in this study. The results of our mediation analysis revealed that emotional intelligence was positively related to psychological well-being. Additionally, the results indicated that self-efficacy and burnout act as partial mediators between emotional intelligence and psychological well-being. As a result, academic administrators and instructors should promote strategies that help athlete–students to gain better emotional intelligence skills, which may also help these students to cope with burnout and increase their self-efficacy, thus increasing their psychological well-being.

## 1. Introduction

According to previous studies, higher education can be defined as a production line, with the output being qualified human resources, thus playing a crucial role in the development of modern society (Barnett, 1992; Trinh, 2023). Students in higher education are generally considered to represent the future of families, societies, and countries (Ansari et al., 2012). Over time, university students have become more representative of wider society, as have their mental health concerns (Topham & Moller, 2011). The years spent by an individual in higher education are generally one of the most important periods of their life, in terms of the development of their physical and mental health and personality. However, this period is also stressful for students—who are in a phase of transition between childhood and young adulthood—as it brings new challenges for them to tackle, such as adaptations to changes in their living conditions, the organization of their social life, taking responsibility for themselves, academic achievement and career expectations, a lack of social interactions, and loneliness (X. Liu et al., 2019; Müller et al., 2022). Therefore, in this phase of young adulthood, students generally experience inner conflicts while dealing with the above-mentioned issues, causing emotional disorders and harm to their mental health (Wang et al., 2022). Taking these factors into account, improving the psychological and mental well-being of higher education students plays a crucial role in their healthy growth and adaptation to society. Thus, it is essential to clarify the underlying mechanisms and factors influencing the psychological and mental well-being of higher education students (X. Liu et al., 2019).

The term psychological well-being (PWB) is defined using a eudaimonic perspective in the study of positive psychology and generally refers to a “generalized feeling of happiness” and absence of mental problems such as depression, stress, and anxiety. With a eudaimonic perspective, Ryff (1989) established a multi-dimensional PWB model in which each dimension describes the different challenges that an individual encounters while trying to function positively, and in combination, these dimensions encompass the scope of wellness (Q. Liu et al., 2009; Ryff & Keyes, 1995). The multi-dimensional PWB model developed by Ryff (1989) and Ryff and Keyes (1995) consists of six elements, which are as follows: (1) self-acceptance (SA)—an individual’s attempts to have positive thoughts about themselves and the ability to be aware of their limitations; (2) positive relations with others (PR)—developing warm and trusting positive social relationships with other individuals; (3) autonomy (AU)—an ability to independently evaluate oneself through personal authority, without looking for approval from other individuals; (4) environmental mastery (EM)—an ability to create or change one’s surrounding environment to make it suitable for one’s personal needs; (5) purpose in life (PL)—individuals who function positively are those who set goals and plan their future by considering their past experiences; and (6) personal growth (PG)—an individual’s ability to realize their own potential and capacity and their desire to keep developing themself.

PWB is an important concept for the prevention of mental health problems in higher education students and the improvement of their adaptation to society. The PWB levels of higher education students have been investigated in many studies in relation to different factors, such as stress and resilience (Freire et al., 2016; Harding et al., 2019; Kotzé & Kleynhans, 2013; Reverté-Villarroya et al., 2021; Sagone & Caroli, 2014; Sugiura et al., 2005), depression and anxiety (Q. Liu et al., 2009), mental toughness (Stamp et al., 2015), mindfulness (Klainin-Yobas et al., 2016; MacDonald & Baxter, 2017), and academic emotions (Kaur et al., 2024).

### 1.1. Emotional Intelligence (EI) and Psychological Well-Being

The concept of EI dates back to the first studies on social intelligence. However, EI gained more attention from researchers in the early 1990s after a study published by two psychologists, Salovey and Mayer (Salovey & Mayer, 1990). Later, the concept was expanded on by Goleman (1995). EI, as a subset of social intelligence, is defined as an individual’s cognitive ability to accurately perceive, understand, and process both their own and other people’s emotions.

As the studies on EI gained more attention from researchers over time, different conceptualizations of EI have emerged. A classification scheme was first proposed by Petrides and Furnham (2000), who classified EI into two distinct categories—ability EI and trait EI—based on the measurement methods used. In particular, ability EI is measured through maximum performance tests, and trait EI is measured using self-reported instruments. This distinction between ability EI and trait EI keeps the development of the research on EI organized and is now accepted as standard in the EI literature produced by researchers (Petrides, 2011). According to MacCann et al. (2022), ability EI is defined as *“knowledge and information processing of emotions and emotion-related knowledge”*, whereas trait EI is a *“set of character traits that underpin social and emotional functioning”*. As pointed out by Petrides (2011) and MacCann et al. (2022), even though both concepts are related to EI, ability EI and trait EI are two distinct concepts with minimal overlap, and the correlations between the measures of the two concepts are very low.

The present study focuses on the trait EI model. As suggested by O’Connor et al. (2019) and Malinauskas and Malinauskiene (2020), when one is interested in measuring behavioral tendencies and self-efficacy, selecting the trait measures of EI is an appropriate choice, as previous studies have shown that the trait EI model is *“a good predictor of coping styles in response to ongoing stressors such as educational contexts”* (O’Connor et al., 2019). Additionally, Laborde et al. (2016) reported that 33 out of 36 identified studies on EI in the sports context conceptualized EI as a trait. In the present study, the EI scale developed by Schutte et al. (1998) was used as the measure of trait EI, as it is a freely available and commonly used reliable measure of trait EI.

Numerous studies have investigated EI in relation to different factors, such as job satisfaction (Othman et al., 2024; Suleman et al., 2020; Tagoe & Quarshie, 2017), academic achievement (Abdolrezapour & Tavakoli, 2012; Fernandez et al., 2012; García-Martínez et al., 2021; Garg et al., 2016), life satisfaction (Blasco-Belled et al., 2020; Kong et al., 2019), and resilience (Schneider et al., 2013; Trigueros et al., 2020).

The literature has indicated that emotionally intelligent people experience a higher level of mental health and PWB than those with a lower level of EI. Some studies have shown that trait EI is an important predictor of PWB (Ahmadi et al., 2014; Carmeli et al., 2009). In the context of higher education students, Urquijo et al. (2016) found that trait EI is positively associated with PWB, and perceived stress acts as partial mediator between EI and PWB among graduate students. A longitudinal study conducted by Malinauskas and Malinauskiene (2020) among male university students reported that perceived social support acts as a partial mediator between trait EI and PWB. Similarly, Zhang et al. (2022) found that social support has a mediating effect between trait EI and well-being. Furthermore, in a recent study by S. Ye et al. (2024), the results of structural equation modeling (SEM) analysis demonstrated that trait EI was positively related to PWB, and positive psychological factors, such as self-efficacy, motivation, and resilience, act as mediators between EI and PWB among Chinese university students.

In the context of physical education and sports, EI plays a vital role in sports performance (Rodriguez-Romo et al., 2021). For instance, in their study comparing ability and trait EI instruments regarding the prediction of sports performance, Kopp et al. (2021) reported that the trait EI instrument (Trait Emotional Intelligence Questionnaire Short Form; TEIQue-SF), developed by Petrides (2009), predicted the self-assessment of athletic performance. Moreover, Ardahan (2012) reported that, among outdoor sport participants, trait EI is positively related with life satisfaction.

### 1.2. Self-Efficacy (SE) and Burnout (BO)

As defined by Bandura (1997), the term self-efficacy (SE) refers to *"beliefs in one’s capabilities to organize and execute the courses of action required to produce given attainments"*. Individuals with a high level of SE experience more positive feelings, which empowers their confidence and motivation, and generally, these highly motivated and confident individuals enjoy life better. Furthermore, when they encounter difficulties, they develop strategies rather than seeing them as personal threats (Klainin-Yobas et al., 2016). Thus, self-efficacy is generally linked to positive well-being, and previous studies have reported that high levels of SE are related to greater PWB (Joekes et al., 2007; Priesack & Alcock, 2015). From the perspective of higher education students, S. Ye et al. (2024) reported that, among university students, trait EI has a significant association with SE, and SE acts as a mediator between EI and PWB.

The concept of self-efficacy in the context of sports was first introduced by D. L. Feltz and Weiss (1982). Self-efficacy in sports is defined as an athlete’s belief in their own abilities to perform performance-related tasks in sports, while athletic self-efficacy describes the athlete’s feeling of being competent and ready to deal with different situations that may arise in sports competitions (Cakiroglu, 2021; Koçak, 2020). D. Feltz et al. (2008) stated that, in terms of sports, the nature of self-efficacy beliefs depends on the research questions of the study and generally involves more complex structures. They classified the self-efficacy beliefs applied to sports into eight different types, as follows: ameliorative and coping efficacy, which is related to athletes’ ability to cope with competition stress or control negative thoughts about their performance; collective efficacy, which is related to a group’s or team’s collective belief in their ability to accomplish a competitive task in sports; competitive efficacy, defined as athletes’ belief in being competitive and successful against opponents; learning efficacy, which is defined as athletes’ belief in their ability to acquire new skills in sports; preparatory efficacy, which is defined as athletes’ efficacy beliefs during preparation for a competition; performance efficacy, which is defined as athletes’ efficacy beliefs in their ability to perform successfully during the competition; self-regulatory efficacy, which is defined as the *“ability to exercise influence over one’s motivation thought processes, emotional states and patterns of behaviour”*; and, finally, task efficacy, which is defined as athletes’ beliefs relating to successfully performing particular tasks with different levels of difficulty.

Self-efficacy is not a unitary construct or trait, and self-efficacy beliefs may vary across various domains (Aerts et al., 2024; Lent, 2020), such as academic self-efficacy, social self-efficacy, career self-efficacy, self-regulatory self-efficacy, and athlete self-efficacy. Physical education and sports in higher education focus not only on advancing theoretical aspects of sports but also on developing practical competitive skills. Therefore, as the target population of the present study is athlete–students studying physical education and sports, the term ‘self-efficacy’ in the present study refers to athletic self-efficacy.

The concept of job burnout was first introduced by Freudenberger (1974), in order to describe physical and mental exhaustion among clinical workers. Later, the concept was further studied and expanded by two social psychologists, Maslach and Jackson (Maslach & Jackson, 1986, 1981). Maslach and Jackson (1986) defined burnout as *"a syndrome of emotional exhaustion, depersonalisation, and reduced personal accomplishment that can occur among individuals who do “people work” of some kind"* and established a multi-dimensional burnout (BO) model consisting of three dimensions: emotional exhaustion, depersonalization, and reduced personal accomplishment. The emotional exhaustion dimension of BO refers to an individual’s feeling of emotional tiredness and being overwhelmed by an excessive workload. The second dimension, depersonalization, refers to a individual’s cynical and negative attitudes towards other people due to their emotional exhaustion. The last dimension, reduced personal accomplishment, refers to an individual’s feeling of being inadequate in given tasks.

In the educational context, the dimensions of BO for students respectively refer to a “student’s feeling of emotional exhaustion”, which occurs due to the excessive course loads and studying, “cynical attitudes” towards learning tasks, which occurs due to academic stress, and a “student’s feeling of being incompetent” due to a lack of academic achievement (Asikainen et al., 2022). BO in higher education students has been investigated by many studies in relation to different factors such as gender (Fiorilli et al., 2022), academic achievement and performance (Cazan, 2015; Kotzé & Kleynhans, 2013; Molero Jurado et al., 2021), social support (Y. Ye et al., 2021), and emotional regulation (Marques et al., 2023). Some researchers have also investigated the relationship between EI and BO among university students; for instance, Loi and Pryce (2022) reported that EI was negatively associated with each dimension of academic burnout. Similarly, Shariatpanahi et al. (2022) reported that medical students with a higher level of emotional intelligence experienced lower levels of academic burnout.

Burnout in the sports setting was first introduced by Caccese and Mayerberg (1984) in their study assessing the levels of burnout in college athletic coaches. The early research on burnout in sport settings was primarily focused on coaches and sport practitioners, using the conceptualization of burnout proposed by Maslach and Jackson (1986). Later, when the scope of the burnout research extended to athletes, the issues concerning the appropriateness and use of Maslach and Jacksons’ conceptualization in athlete burnout studies eventually led to the development of athlete-specific burnout measures (Goodger et al., 2007). Raedeke and Smith (2001) defined athlete burnout as *“a psychological syndrome of emotional/physical exhaustion, reduced sense of accomplishment and sport devaluation,”* and developed an Athlete Burnout Scale composed of three dimensions": *“emotional/physical exhaustion,”* which is related to the intense demands of sports training and competition; *“reduced sense of accomplishment,”* which is related to unmet expectations and inabilities to reach a goal in sports; and, finally, *“sport devaluation,”* which is related to athletes’ uncaring behaviors about sports and their own performance.

In the context of athlete–students, burnout is one of the most common mental health problems. As athlete–students deal with academic courses and athletic training in parallel, this dual demand for success in both areas may result in burnout and decrease both the academic and athletic performance of athlete–students. Sports, by nature, involves competition, and athletes are generally driven by sport achievements, which requires dedication and high levels of stress (Lee et al., 2017). Previous studies have shown that burnout in athlete–students is negatively associated with their life satisfaction and psychological well-being (Gabana et al., 2017; Gerber et al., 2018; Lee et al., 2017). Moreover, Glandorf et al. (2023) showed that athlete burnout was associated with increased negative mental health outcomes and decreased positive mental outcomes. Regarding Turkish athlete–students, Doğan (2019) reported that athlete–students in physical education and sports fields experience high levels of burnout. In the present study, as the target population is athlete–students studying physical education and sports, the term ‘BO’ refers specifically to athlete burnout.

### 1.3. The Present Research

Despite the significant amount of previous work on the relationship between EI and PWB, the research on physical education and sports students remains limited. Likewise, studies conducted in Türkiye are also limited. Moreover, to the best of our knowledge, no previous research has focused on jointly incorporating the above-mentioned constructs in the context of physical education and sports athlete–students. Thus, based on the above-mentioned theoretical research and literature review, the present study aims to achieve the following:

- Investigate the relationship between EI and PWB among Turkish physical education and sports athlete–students;
- Investigate whether SE and BO act as mediators in the relationship between EI and PWB among athlete–students.

**Figure 1:**
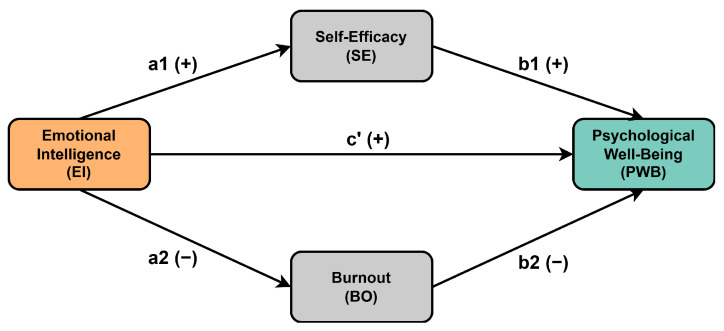
Hypotheses of the research model.

Therefore, the six following hypotheses were proposed (which are also summarized in Figure 1):

- $H_{1}$: EI is positively related to PWB;
- $H_{2}$: EI is positively related to SE;
- $H_{3}$: EI is negatively related to BO;
- $H_{4}$: SE is positively related to PWB;
- $H_{5}$: BO is negatively related to PWB;
- $H_{6}$: SE and BO mediate the relationship between EI and PWB.

## 2. Materials and Methods

### 2.1. Participants and Procedures

The sample consisted of 226 (163 undergraduate and 63 graduate) athlete–students from Osmaniye Korkut Ata University, School of Physical Education and Sports. Among the participants, 38.5% (*n* = 87, undergraduate = 72, graduate = 15) were categorized as female, and 61.5% (*n* = 139, undergraduate = 91, graduate = 48) were categorized as male; furthermore, 72.1% of the participants were between the ages of 18 and 27.

This cross-sectional study was conducted in Osmaniye Korkut Ata University, School of Physical Education and Sports, with the ethical approval of the ethics committee on health sciences research of the Osmaniye Korkut Ata University (Registration no: E.202521, Date: 1 November 2024, Decision No: 2024/6/3). Initially, all students were informed about the contents of the questionnaire, and those who agreed to participate in the study completed an informed consent form.

### 2.2. Instruments

#### 2.2.1. Psychological Well-Being Scale (PWBS)

In the present study, the Turkish version of the PWBS scale adapted by Telef (2013) was used to assess PWB. The PWBS scale was originally developed by Diener et al. (2010) and consists of eight short items as a summary survey of factors including self-esteem, optimism, relationships, purpose, and meaning. The items are rated on a 7-point Likert-type scale ranging from 1 (strong disagreement) to 7 (strong agreement). The PWBS scale provides a single score for PWB, yielding an overview of PWB across diverse domains. The minimum and maximum scores that can be obtained from the PWBS scale are 8 and 56, respectively. All items of the scale are phrased in the positive direction. In the present study, the Cronbach’s alpha statistic was used to examine the internal consistency of the scale, where the Cronbach’s α value was 0.92.

#### 2.2.2. Emotional Intelligence Scale (EIS)

The EIS scale was originally developed by Schutte et al. (1998) as a 33-item scale, in order to measure the emotional intelligence levels of indviduals. The scale was based on the emotional intelligence model developed by Salovey and Mayer (1990). Later, Lane et al. (2009a) investigated the factorial validity of the scale for use in sports studies and constructed a 19-item EI scale. The scale consists of 5 dimensions, as follows: appraisal of others’ emotions, appraisal of own emotions, regulation, social skills, and utilization of emotions. The items of scale are rated on a 5-point Likert-type scale with values ranging from 1 (strongly disagree) to 5 (strongly agree). In this study, the Turkish version of the EIS scale developed by Lane et al. (2009a) was used to assess EI. The adaptation of the scale to Turkish, as well as validity and reliability tests, were performed by Adiloğulları and Görgülü (2016). One item in the “appraisal of others’ emotions” sub-dimension with insufficient factor loading was dropped, resulting an 18-item EIS scale. In the present study, the Cronbach’s alpha statistic was used to examine the internal consistency of the scale, where the Cronbach’s α value was 0.86.

#### 2.2.3. Athlete Self-Efficacy Scale (ASES)

The Athlete Self-Efficacy Scale (ASES) scale was developed by Koçak (2020) as a 16-item scale, aiming to measure the SE of individuals who participate in sports and athletes. The scale consists of 4 dimensions—namely, sport discipline efficacy, psychological efficacy, professional thought efficacy, and personality efficacy—with items rated on a 5-point Likert-type scale ranging from 1 (I do not agree) to 5 (I agree completely). The minimum and maximum scores that can be obtained from the ASES scale are 16 and 80, respectively.

As the study of physical education and sports involves physical exercise and competition, the term SE in sports generally involves a more complex structure of SE, including dimensions such as collective efficacy, competitive efficacy, learning efficacy, and performance efficacy (D. L. Feltz & Weiss, 1982; Koçak, 2020). Therefore, in the present study, the ASES scale was utilized in order to measure the SE levels of students. In the present study, the Cronbach’s alpha statistic was used to examine the internal consistency of the scale, where the Cronbach’s α value was 0.92.

#### 2.2.4. Athlete Burnout Questionnaire (ABQ)

The Athlete Burnout Questionnaire (ABQ) was originally developed by Raedeke and Smith (2001) as a 15-item scale, in order to measure the BO levels of individuals who participate in sports and athletes. The scale consists of 3 dimensions—namely, emotional/physical exhaustion, devaluation, and reduced sense of accomplishment—with items rated on a 5-point Likert-type scale with values ranging from 1 (almost never) to 5 (almost always). In this study, the Turkish version of the ABQ adopted by Kelecek et al. (2017) was used to assess BO. While the Turkish version of the ABQ scale consists of 13 items, 2 items (i.e., item 1 and item 11) were dropped due to the low factor loadings. In the present study, the Cronbach’s alpha statistic was used to examine the internal consistency of the scale, where the Cronbach’s α value was 0.86.

### 2.3. Statistical Analysis

The data used in the present study were analyzed using the Statistical Package for Social Sciences software (SPSS v.25). For data analysis, a three-step approach was followed. In the first step, the normality of the data was investigated using Kolmogorov–Smirnov tests, histograms, and Q-Q plots. In the second step, descriptive statistics related to EI, SE, BO, and PWB were calculated. Additionally, as the results of the Kolmogorov–Smirnov tests concluded that the EI, SE, BO, and PWB did not follow a normal distribution (*p* < 0.05) the non-parametric Spearman’s rank correlation coefficient was used to evaluate the associations between variables. In the third step, mediation analysis was performed with a multiple mediation model containing two parallel mediators (Hayes model no. 4) using the SPSS PROCESS Macro (version 4.2) developed by Hayes (2022). In the model, EI was the independent variable, PWB was the dependent variable, and SE and BO were the mediators. Additionally, a bootstrapping methodology proposed by Hayes (2022) was also utilized in order to test the statistical significance of the mediating effects. The bootstrap sample consisted of 5000 cases, and the confidence interval was set at 95%.

## 3. Results

### 3.1. Common Method Bias Test

In this study, Harman’s single factor test was performed in order to identify the common method bias (CMB) caused by the data collection approach (questionnaire). The results of Harman’s single factor test showed that there were 13 factors with eigenvalues greater than 1, and the variance explained by the first factor was 25.43%, which was less than 50%. Therefore, it can be said that CMB did not affect the results of the data analysis.

### 3.2. Descriptive Statistics and Correlation Analysis

The Spearman correlation coefficients and descriptive statistics of variables are presented in Table 1. According to the table, emotional intelligence was significantly positively correlated with psychological well-being (r=0.55, p<0.01) and was significantly positively correlated with self-efficacy (r=0.57, p<0.01). Self-efficacy was significantly positively correlated with psychological well-being (r=0.55, p<0.01). Additionally, statistically significant moderate negative correlations were found between emotional intelligence and burnout (r=−0.31, p<0.01) and between burnout and psychological well-being (r=−0.40, p<0.01). The above-mentioned statistically significant correlations that were found between the variables of the model proposed in Figure 1 provided a good rationale for subsequent mediation analysis.

### 3.3. Mediation Analysis

**Figure 2:**
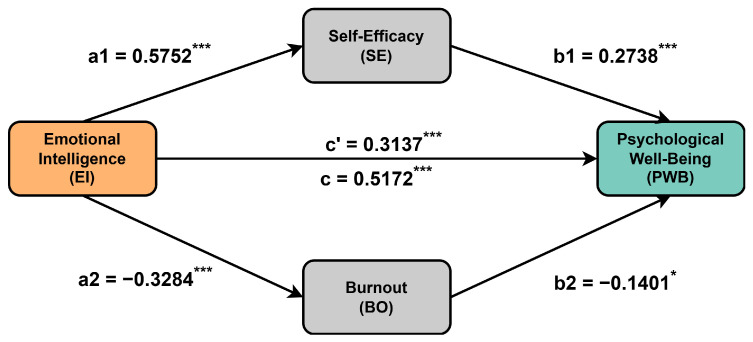
Parallel mediation model of self-efficacy and burnout in the relationship between emotional intelligence and psychological well-being. Standardized beta coefficients are presented. (c’) and (c) indicate direct and indirect effects, respectively. ^∗∗∗^ p<0.001, ^∗^ p<0.05.

The results of parallel mediation analysis are given in Figure 2 and Table 2. As shown in Figure 2, in path a1, EI significantly positively predicted SE (β=0.5752, t=10.5242, p<0.001) while, in path a2, EI significantly negatively predicted BO (β=−0.3284, t=−5.2035, p<0.001), thus confirming Hypothesis 2 (EI is positively related to SE) and Hypothesis 3 (EI is negatively related to BO). Furthermore, the results indicated that SE positively predicted PWB (path b1; β=0.2738, t=3.9269, p<0.001) and BO negatively predicted PWB (path b2; β=−0.1401, t=−2.3199, p<0.05). These findings confirm Hypothesis 4 (SE is positively related to PWB) and Hypothesis 5 (BO is negatively related to PWB). Finally, EI significantly positively predicted PWB (path c’; β=0.3137, t=4.7347, p<0.001), which confirms Hypothesis 1 (EI is positively related to PWB).

According to Table 2, which shows the results of the mediation analysis, the total indirect effect (Effect=0.2035, BootSE=0.0657, 95%CI(BootLLCI=0.0800,BootULCI=0.3388)) accounted for 39.35% of the total effect (Effect=0.5172, BootSE=0.0553, 95%CI(BootLLCI=0.3913,BootULCI=0.6093)), and the 95% CIs did not contain zero. This indicates that SE and BO act as partial mediators between EI and PWB, thus confirming Hypothesis 6 (SE and BO mediate the relationship between EI and PWB). Additionally, the mediating effect of SE between EI and PWB (indirect path 1: EI→SE→PWB) accounted for 30.45% of the total effect, while the mediating effect of BO between EI and PWB (indirect path 2: EI→BO→PWB) accounted for 8.90% of the total effect. The 95% CIs of indirect path 1 and indirect path 2 did not contain zero.

## 4. Discussion and Implications

The aim of the present study was to investigate the relationship between EI and PWB among athlete–students in higher education, specifically targeting athlete–students in the physical education and sports discipline through exploring the potential impacts of SE and BO on the relationship between EI and PWB. The findings of the study were in line with the proposed model, confirming all the hypotheses of the study. One major finding of the mediation analysis was that EI was a positive predictor of PWB and SE and a negative predictor of BO. The second major finding was that SE and BO both act as partial mediators in the relationship between EI and PWB among higher education students. The results of the mediation analysis indicated that students in higher education with high levels of EI experience high level of PWB, which is in line with the previous literature. For instance, in a recent study conducted among university students, Kartol et al. (2024) reported that EI was a positive predictor of life satisfaction, which is an important factor in mental health and psychological well-being. Similar results have also been reported by Göçet Tekin (2021) and Zhang et al. (2022) in the context of higher education students. In athlete–students, in line with the findings of the present study, the results of the study conducted by Malinauskas and Malinauskiene (2018) among male university athletes students revealed that trait EI is positively related with PWB. Similar results have also been reported by Lane et al. (2009b) in their study on athlete–students. One other finding of the present study was that EI was a positive predictor of SE and a negative predictor of BO; this finding is also consistent with previous studies. For instance, in their study conducted among university students, Chang and Tsai (2022) reported that students with a higher level of EI tend to have higher self-efficacy and learning motivation. Similarly, the results of the study by Sun and Lyu (2022) showed that EI in college students has a direct positive effect on self-efficacy. In their longitudinal study conducted among higher education students and teachers, Leonova et al. (2021) concluded that EI has both direct and indirect effects on burnout. They reported that the direct effect involved the prevention of emotional exhaustion through the regulation of negative emotions, while the indirect effect was mediated through self-efficacy. They also added that the protective effect of EI on the prevention of BO is evident in stressful conditions characterized by heavy workloads. This finding is also in line with the results of studies in athlete–student and sports contexts. For instance, Tutal and Efe (2020) reported that trait EI is an important determinant of athlete self-efficacy in athlete–students, and as the trait EI increases, the athletic self-efficacy levels of athlete–students also increase. In a recent study conducted by Zajonz et al. (2024), the results indicated that trait EI is negatively associated with athlete-burnout in athlete–students.

Regarding the results of the mediation analysis, two indirect paths reflecting the relationship between EI and PWB were investigated. In indirect path 1, SE mediated the relationship between EI and PWB. The mediating effect of SE on the relationship between EI and PWB has also been reported in a recent study by S. Ye et al. (2024), which was conducted among university students. As for indirect path 2, BO mediated the relationship between EI and PWB. This finding is also in line with the previous literature; for instance, the results of the longitudinal study by Carvalho et al. (2018) demonstrated that EI promoted life satisfaction over time among health students, and burnout indirectly explained the relationship between EI and life satisfaction. Similar results have also been reported in a previous study by Cazan and Năstasă (2015), conducted among university students in Romania.

Previous studies have shown that athletes with higher levels of EI are more successful in managing their emotions, and engaging in adaptive coping strategies such as relaxation techniques may also help them to be resilient to stress and burnout (Zajonz et al., 2024); along these lines, the present study demonstrated that trait EI has a positive effect on PWB among higher education athlete–students, and SE and BO act as mediators between EI and PWB.

The results of the present study have significant practical implications for educational psychologists, sport psychologists, and academic administrators and instructors, as they provide information regarding the PWB of higher education athlete–students through the investigation of several underlying influencing factors such as EI, SE, and BO.

Due to the influence of EI on different constructs such as psychological well-being, academic performance, and work performance, EI training through interventions aiming to improve different outcomes, such as life satisfaction, self-efficacy, and mental health, has recently gained more attention (Hodzic et al., 2018); for instance, in the context of athlete–students, the results of the study conducted by Shahbazzadegan et al. (2013) among female running athlete–students indicated that, after 8 weeks of educational sessions focused on EI and positive thinking, significant increases in mental health and self-efficacy levels were observed.

Training EI in athlete–students improves not only their self-efficacy and athletic performance levels but also may help them to engage in stress-coping strategies to reduce their burnout levels and increase their mental health and life satisfaction. Therefore, the findings of the present study are expected to help academic administrators and instructors in developing better strategies to improve the PWB levels of athlete–students, such as including courses on positive psychology into their curriculum to improve the emotional intelligence skills of student–athletes.

## 5. Limitations and Future Research

The present study has several limitations. As a first limitation, the study was designed as a cross-sectional study, and the data were collected within a certain time period, thus making it impossible to draw conclusions regarding casual relationships between the variables. Thus, further longitudinal studies are needed to validate the effects reported in this study. The second limitation is that the study did not account for the socio-economic status of the students in the data analysis, which is also closely linked with PWB. The third limitation is that, as the study was conducted among Turkish athlete–students and the measures for SE and BO used in the study are specific to athletes, generalization of the results may be restricted for all higher education students in different professions and those residing in different nations. Finally, although the sample size for the present study was adequate to provide enough statistical power, a larger sample size was needed to analyze the sub-scale dimensions of EI, SE, and BO in the parallel mediation analysis.

Despite its limitations, the present study provides preliminary evidence for the mediating roles SE and BO in the relationship between EI and PWB in athlete–students. The findings of the study may serve as a starting point for future research on the underlying mechanisms of the relationship between EI and PWB; for instance, as mentioned in previous sections, athlete–students commonly deal with the dual demands of success both in academic courses and athletic trainings. This situation may become a source of chronic stress and increase the risk of burnout, which may negatively affect the mental health and PWB of athlete–students. On the other hand, EI promotes the effective regulation of emotions, which is closely linked to both BO and the ameliorative and coping efficacy of athletes. Therefore, regarding the role of EI in promoting the effective regulation of emotions, a deeper understanding of the relationships between EI, SE, and BO may help to better understand the relationship between EI and PWB in athlete–students.

Additionally, EI promotes stronger social networking and participation among students through encouraging collaboration, fostering effective communication, and enabling adaptation in students from diverse cultural and ethnic backgrounds (Zhoc et al., 2020). From the athlete–student perspective, a stronger social network and social participation may increase the collective efficacy levels of athlete–students, thus resulting in higher sports performance and PWB.

Finally, the trait EI model used in this study conceptualizes EI in a manner similar to stable personality traits and is more resistant to change than ability EI models, which are based on set of skills that can be learned and, thus, are more flexible with respect to development (Hodzic et al., 2018). Moreover, the multi-level meta-analysis of EI training conducted by Hodzic et al. (2018) confirmed that training based on ability EI is much more effective than that based on trait EI. Thus, future research on the relationship between EI and PWB among athlete–students may consider the usage of different EI models, such as ability EI, as well as the further incorporation of the EI, SE, and BO sub-dimensions.

## 6. Conclusions

The improvement of higher education athlete–students’ mental health and PWB is important for their healthy growth and adaptation to college life and society. Thus, it is essential to clarify the influencing factors of PWB among higher education athlete–students. The findings of the present study demonstrated that EI has a significant positive effect on PWB among higher education athlete–students, and SE and BO mediate the relationship between EI and PWB. Previous studies have examined the relationship between EI and PWB including SE and BO individually; however, to the best of our knowledge, no previous research has focused on incorporating the SE and BO constructs together to investigate the relationship between EI and PWB among higher education athlete–students.

## Figures and Tables

**Table 1:** Descriptive statistics and Spearman correlation coefficients for scales (N = 226).

Variables	Score Ranges	Mean	S.D.	1	2	3	4
1. EI	18–90	68.85	8.68	1			
2. SE	16–80	62.95	10.02	0.57 **	1		
3. BO	13–65	22.24	8.89	−0.31 **	−0.38 **	1	
4. PWB	8–56	44.54	8.39	0.55 **	0.55 **	−0.40 **	1

** p<0.01. EI: emotional intelligence; SE: self-efficacy; BO: burnout; PWB: psychological well-being.

**Table 2:** Mediation Analysis of EI and PWB (N = 226).

			95% Confidence Interval		
	Effect	BootSE	BootLLCI	BootULCI	Relative Mediation Effect
Total effect	0.5172	0.0553	0.3913	0.6093	100%
Direct effect	0.3137	0.0641	0.1771	0.4297	60.65%
Total indirect effect	0.2035	0.0657	0.0800	0.3388	39.35%
Indirect path 1: EI→SE→PWB	0.1575	0.0621	0.0412	0.2876	30.45%
Indirect path 2: EI→BO→PWB	0.0460	0.0224	0.0059	0.0951	8.90%

*EI*: emotional intelligence; *SE*: self-efficacy; *BO*: burnout; *PWB*: psychological well-being. Effects are standardized. Bootstrap sample size: 5000. The 95% CIs do not contain zero.

## Data Availability

The data presented in this study are available on request from the corresponding author. The data are not publicly available due to ethical reasons.

## Abbreviations

The following abbreviations are used in this manuscript:

EI	Emotional Intelligence
SE	Self-Efficacy
BO	Burnout
PWB	Psychological Well-Being
